# Application of Locked Nucleic Acid Oligonucleotides for siRNA Preclinical Bioanalytics

**DOI:** 10.1038/s41598-019-40187-4

**Published:** 2019-03-05

**Authors:** Mai B. Thayer, Julie M. Lade, David Doherty, Fang Xie, Babak Basiri, Omar S. Barnaby, Noor S. Bala, Brooke M. Rock

**Affiliations:** 1 Amgen Research, Pharmacokinetics and Drug Metabolism, South San Francisco, 94080 USA; 2 Amgen Research, Pharmacokinetics and Drug Metabolism, Thousand Oaks, 91320 USA

## Abstract

Despite the exquisite potential of siRNA as a therapeutic, the mechanism(s) responsible for the robust indirect exposure-response relationships have not been fully elucidated. To understand the siRNA properties linked to potent activity, requires the disposition of siRNA to be characterized. A technical challenge in the characterization is the detection and quantitation of siRNA from biological samples. Described herein, a Locked Nucleic Acid (LNA) Hybridization-Ligation ECL ELISA was designed for ultra-sensitive quantification of both sense and antisense strands of siRNA independent of structural modifica-tions. This assay was applied to measure siRNA in serum and tissue homogenate in preclinical species. We observed rapid clearance of siRNA from the systemic circulation which contrasted the prolonged accumulation within the tissue. The assay was also able to distinguish and quantify free siRNA from RNA-induced silencing complex (RISC) and Argonaute 2 (Ago2) associated with therapeutic siRNA. We utilized an orthogonal method, LC-MS, to investigate 3′ exonuclease activity toward the antisense strand metabolism. Taken together, we have demonstrated that the LNA Hybridization-Ligation ECL ELISA is arobust analytical method with direct application to measuring the exposure of siRNA therapeutics seamlessly across biological matrices.

## Introduction

Over the past two decades RNA interference (RNAi) has emerged as a powerful route for silencing gene expression^1^. In 1998, the term RNAi was coined referring to the phenomenon of post-translational silencing of gene expression that occurs in response to the introduction of double-stranded RNA (dsRNA) into a cell^2^. In 2006, Andrew Z. Fire and Craig C. Mello were awarded the Nobel Prize for their discovery of RNAi gene silencing by double-stranded RNA^3^. The RNAi field quickly expanded with new feats in gene expression knockdown in cell culture^4^. RNAi technology advancements can now be exploited to allow specific functional inhibition of almost any chosen target gene, allowing much more rapid functional genetic characterization.The natural mechanism of RNA inhibition is mediated by small, double-stranded RNA molecules of 19–25 nucleotides. It is generally accepted that the RNAi function through a multi-step mechanism^5^. Upon entrance to the cell, longer double-stranded RNA molecules are first processed by the RNAse enzyme, Dicer^6–8^. This functional dimer contains helicase, dsRNA binding, and PAZ (named after piwi, argonaute, and zwille proteins) domains^9^. The former two domains are important for double-stranded RNA unwinding and facilitation of RNA interactions. The function of the PAZ domain is not fully understood^10^. Dicer produces 21–23 nucleotide RNA fragments usually with two nucleotide 3′ end overhangs, which are termed siRNA (silencing RNA). The silencing mechanism of siRNA is mediated through the RNA-induced silencing complex (RISC) which, guided by the 21-23 nucleotide fragments (siRNA), recognizes complementary sequence resulting in cleavage and degradation of the targeted mRNA^11^. With this mechanism, gene expression is specifically inactivated at a post-transcriptional level.

Recent advances with RNAi and siRNA synthetic chemistry have fueled interest in therapeutic siRNA molecules. Antisense oligonucleotides (ASOs) have made landmark achievements in the treatment of several diseases including neurological disorders^12^, ocular disease^13^, and early cardiovascular disease^14^. Synthetic siRNAs have been demonstrated to target genes *in vivo* for multiple disease areas including cancer^15–17^ hypercholesterolemia^18^, liver cirrhosis^19^, respiratory syncytial virus^20^, hepatitis B^21^ and human papillomavirus^22^. Numerous synthetic siRNAs are under development for various diseases. As of August 2018, the first siRNA therapeutic received approval^23^. With additional clinical trials, it is anticipated that more nucleic acid-based therapeutics will soon reach the market.

Improvements in delivery and chemical stability have significantly improved tissue-specific targeting, cell entry, and sustained potency of siRNA therapeutics. The combined advancements have had dramatic impact on efficacy, thereby decreasing the effective doses. To adequately address the exposure-response relationships in complex matrices, the bioanalytical method platform needs to be carefully selected based on the structure and function of the therapeutic candidate. Quantitative, highly specific and sensitive methods are a requirement to determine the pharmacokinetic (PK) parameters and pharmacodynamic (PD) relationships, both in drug discovery as well as in the drug development process of an siRNA therapeutic.

Several methods for quantifying siRNA in mammalian cell lines and *in vivo* applications have been reported. Various PCR-based siRNA detection methods have been developed, including primer-extension RT-PCR^24^, stem-loop RT-PCR^25^, and competitive quantitative PCR^26^. These methods, however, suffer from time-consuming and costly optimization processes. Singh *et al*. were the first to describe the Locked Nucleic Acid (LNA) analogs which display unprecedented affinity towards both complimentary DNA and RNA^27^. It has been demonstrated that the incorporation of a single LNA can greatly increase duplex stability with pyrimidine interactions^28^. Here, we developed an ultra-sensitive ELISA-based assay for quantifying double-stranded intact siRNAs for *in vivo* pharmacokinetic analysis. The assay makes use of two distinct locked-nucleic acid (LNA) modified DNA probes with 5′ and 3′ labeling with biotin (capture marker) and digoxygenin (detection marker), respectively. The assay provides an easy-to-use procedure while delivering high sensitivity and double-stranded siRNA specificity from biological samples. The utility of the ELISA-based assay is demonstrated through the quantitation of intracellular siRNA and *in vivo* serum pharmacokinetics, allowing the efficacy of chemical modifications and various delivery systems to be readily assessed.

## Results and Discussion

### Design of LNA Hybridization-Ligation ECL ELISA

The overall design of the hybridization oligonucleotide sandwich was modified from a previously published ELISA-based assays^29,30^. Summarizing previous assay designs, two DNA-based oligonucleotide probes are implemented. One acts as a capture with its 3′ biotin and 5′ 9-mer overhang and the other acts as a detection probe with its complimentary bases to the capture overhang and 3′ digoxygenin attachment. In previously reported work, the assay structure follows a two-step hybridization-ligation design. Briefly, the analyte of interest is denatured and hybridized in solution with the capture probe and is subsequently attached to a plate via avidin-biotin association. Next, the detection probe is mixed with a T4 ligation mix and after incubation is washed to remove any non-ligated detection probe. The samples are then treated with S1 nuclease in a sodium chloride solution to cleave any truncated duplex. After blocking, their assay detects signal with an anti-digoxigenin-AP substrate solution and is read on a fluorescence plate reader.

**Figure 1:**
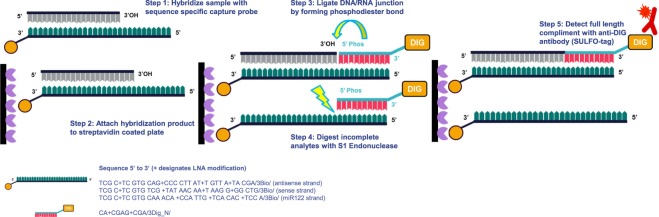
Illustration of the hybridization-ligation ECL ELISA including sequences used for the biotinylated and digoxygenin conjugated probes.

Here we have demonstrated significant assay improvements with the application of LNA chemical base modifications throughout the assay probes, re-tooling of the enzymatic reactions, and transferring the assay to an electro-chemiluminescent (ECL) platform. Figure 1 displays the assay schematic and Table 1 summarizes the improvements between previously published assays and the LNA applied ELISA reported here.

**Table 1:** Summary of differences between assays previously published to new assay incorporating LNA nucleotide modifications.

Assay Parameters	Wei *et al*.^29^, Chan *et al*.^30^	LNA Hybridization-Ligation ECL ELISA
Probe concentrations	200 nM capture	50 nM capture
	100 nM detection	1 nM detection
T4 ligase conditions	18 °C, overnight	37 °C, 1 hr
S1 nuclease conditions	NaCl buffer (pH 8.0), 60 U/well 25 °C, 2.5 hrs	ZnSO4 + Nacl Buffer (pH 5.4), 40 U/well 25 °C, 30 mins
Assay Format	Fluorescence ELISA	Electro-chemiluminescent ELISA
Total assay time	48 hrs	4 hrs
LOD (serum)	10 pM	1.0 pM
LOD (liver/kidney)	No assay reported	1.0 pM

In designing optimized probes for quantification of double-stranded siRNA, LNA substitutions were interspersed into the capture and detection probes targeting a theoretical melting temperature (Tm) of 80 °C and 69 °C, respectively. It is important to note that LNA substitutions adjacent to the nucleation cut site were avoided to allow for S1 nuclease enzyme accessibility. Further, taking into consideration that the optimal hybridization temperature for LNA-containing probes is 30 degrees below the theoretical Tm, a final hybridization temperature of 52 °C was ultimately empirically determined, reflecting LNA oligonucleotide manufacturer’s recommendation of 30 degrees below theoretical Tm. As anticipated, the directed incorporation of LNA into a DNA oligomer dramatically increased probe affinity to the siRNA analyte, therefore, reducing the final assay concentrations of capture and detect probes by 4- and 100-fold, respectively, in comparison to previously published methods. In addition to increasing the affinity of the hybridization probe to the analyte of interest, an increase in thermo-stability was also gained from LNA substitution into the digoxygenin-conjugated detection probe. Moreover, a typical ligation reaction for double stranded DNA is carried out overnight at 18 °C, whereas we were able to decrease this reaction to an hour incubation at 37 °C, thereby, further minimizing the total assay duration. Moving beyond capture and detection probe optimization, further assay improvements included identification of ZnSO4 as an ideal cofactor for achieving maximal S1 nuclease activity. For analyte spikes in buffer and serum, we observed peak nuclease activity under the following reaction conditions: pH 5.5 at room temperature for 30 minutes. For more complex matrices, such as tissue homogenates, we found a dramatic improvement in the signal-to-noise ratio by increasing the incubation temperature from 25 °C to 29 °C. The impact of both metal cofactor and pH on assay performance are summarized in Supplemental Information.

**Figure 2:**
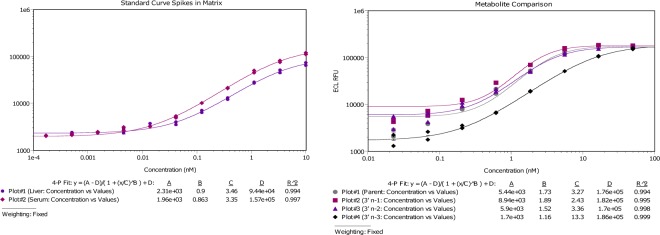
LNA Hybridization-Ligation ECL ELISA assay sensitivity and specificity. (**A**) The assay demonstrates pM sensitivity in both rat serum and liver homogenate (n = 2). The lower limit of quantification with this assay in both serum and liver is 1.0 pM. (**B**) The assay is selective for both 3′ n-1 and n-2 metabolites with a response of 133% and 95% reactivity when comparing the EC50 values back to the intact siRNA molecule (n = 2). The 3′ n-3 metabolite displayed a right-shifted EC50 with a reactivity of 25% compared to full-length siRNA molecule (n = 2). Graphs fitted in SoftMax Pro (Molecular Devices, LLC. San Jose, CA) with a 4-parameter fit with fixed weighting.

Lastly, to enhance the assay’s overall dynamic range, we transferred the method from a standard fluorescent plate readout to the Meso Scale Discovery (MSD) ECL platform. An anti-digoxygenin detection antibody conjugated to a ruthenium (II) tris-bipyridine N-hydroxysuccinimide tag was incubated with samples following the nuclease reaction. In a direct comparison of DNA versus LNA probes with an anti-digoxygenin detection, we found that the addition of LNA modification dramatically increased sensitivity (See Supplemental Information). The combined assay design modifications produced an assay with a dynamic range of 0.1–10,000 pM in serum, liver and kidney homogenate with a level of detection (LOD) of 1.0 pM (Fig. 2). Most impressively, the re-tooled assay enables the measure siRNA molecules from liver homogenate in a much shorter time frame, with no change to the assay sensitivity (Table 1). Due to the duplex nature of siRNA molecules and the denaturing conditions of this assay, probes can be designed specifically to the sense or antisense strands. Although there are methods for the characterization of duplex RNA such as native Mass Spectrometry (MS)^31^, these methods are not nearly as sensitive enough to employ in the quantification of siRNA *in vivo*. For our testing of either strand, samples are homogenized and two assays for sense and antisense are run in parallel. In this manner, we conclude that overlaying values correspond to siRNA in duplex form, and a divergence in values corresponds to a single-stranded population.

### Specificity of LNA Probes

Chemical modifications made to the ribose or phosphate backbone are now routinely incorporated into siRNA molecules in an effort to mitigate exo- and endo-nuclease activity within the blood and target tissue compartments. That being said, it is also well established in the literature that even fully chemically modified oligonucleotides are still subjected to nucleolytic activity *in vivo*^32^. In line with this, the specificity of our LNA capture probes were tested in the presence of synthetic metabolites created to mimic exonucleolytic degradation of the antisense strand of the siRNA duplex. These metabolites contain 2′-O-methyl chemical modifications on the ribose across all nucleotides in order to test the extent of probe cross-reactivity toward truncated oligo species. We incorporated this chemical modification for the sole purpose of gaining stability in solution. Previously published efforts using an *ex vivo* rat liver homogenate model demonstrated that 3′-exonucleases were predominantly responsible for mediating antisense oligonucleotide metabolism relative to either 5′-exonuclease or endonuclease activity; therefore, our work focused on LNA probe cross-reactivity with metabolites that resulted from 3′ end clipping of the antisense strand^33^. To ensure proper translation of rodent to NHP exonuclease activity, we assessed *in vitro* samples and saw clear translation of 3′-exonuclease activity, with minimal 5′-metabolites formed (See Supplemental Information for rat data). Both 3′ n-1 and n-2 metabolites (designated as one and two nucleotide subtractions, respectively) demonstrated an assay response in strong agreement with the full-length antisense strand, with EC50 values of 2.43 nM (1.3-fold of antisense strand) and 3.36 nM (0.97-fold of antisense strand), respectively, whereas 3′ n-3 exhibited a dramatic right-shifted response with an EC50 of 13.3 nM (0.25-fold of antisense strand) (Fig. 2). It is important to note that because cross-hybridization of the LNA probe was observed with 3′ n-1 and n-2 metabolites, it is possible that exclusive quantitation of the full-length antisense strand may be overestimated using this method. Regardless, LNA ELISA is able to capture a total population of full-length antisense, 3′ n-1 and n-2, of plausibly active species as it has been demonstrated that siRNA truncated species can still effectively load the RNA-induced silencing complex and mediate potent mRNA target knockdown^34^.

### Characterization of antisense strand metabolites formed *in vivo* using LC-MS

Antisense oligonuceotide therapeutics are reported to undergo biotransformation in plasma^35^; however, unlike small molecule drugs which undergo biotransformation by phase I and phase II drug metabolizing enzymes^36^, siRNA biotransformation occurs by clipping of the phosphodiester bonds between nucleic acids or by innate chemical instability. Numerous reports of oligonucleotide metabolism have been described^32,37^, and have largely employed ion-pair reversed-phase chromatography coupled to ion trap mass spectrometers. More recently, high-resolution mass spectroscopy has been utilized for the identification of metabolites from *in vitro* matrices.

**Figure 3:**
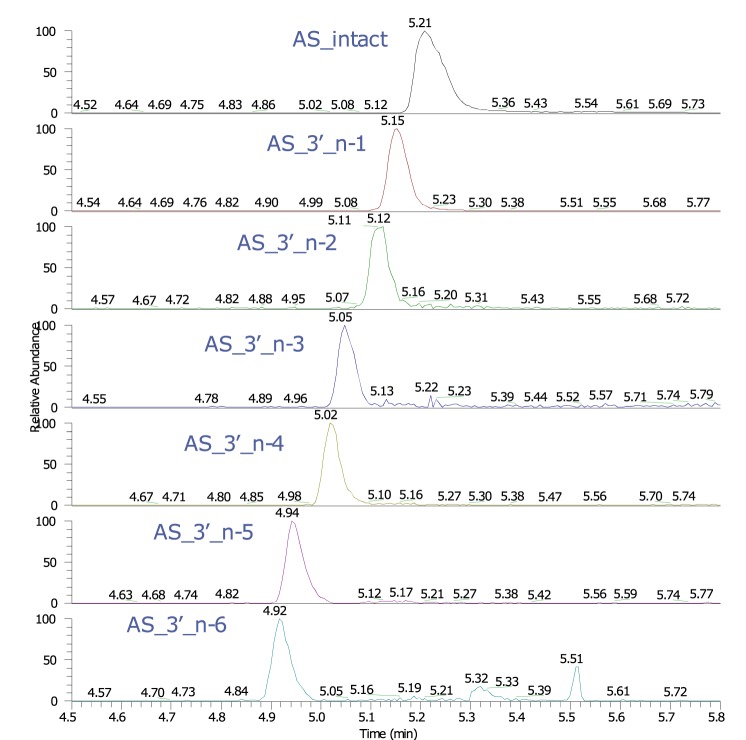
Extracted ion chromatogram of the full length antisense strand and metabolites in NHP liver. With LC-MS analysis, we observed the full siRNA antisense strand and down to six nucleotide base subtractions from the 3′ end.

In this work, we applied high-resolution accurate-mass measurements to elucidate the metabolites from liver and kidney tissue of non-human primates (NHPs). The application of accurate-mass ion detection affords a high level of confidence in the metabolite identification. In an exploratory NHP study, animals were administered a subcutaneous injection of a proprietary siRNA once weekly for four weeks at a dose of 300 mg/kg. A representative extracted ion chromatogram resulting from the LC-MS data of the samples from non-human primate liver homogenate is shown in Fig. 3. The sequential degradation pattern suggests that these metabolites are the result of 3′ exonuclease activity upon the antisense strand of the duplex. Currently, little is known about the types of RNAses and/or nucleases involved in the degradation of the synthetic siRNAs, and this is an active area of research in our laboratory. Table 2 lists the percentage of intact antisense strand and metabolites in NHP liver and kidney samples. In the NHPs the major species observed in tissues is the intact antisense strand. This ensures that the assay is in line with the observed catabolites that were measured *in vivo* by LC/MS.

**Table 2:** Percentage of intact antisense strand and metabolites in NHP liver and kidney samples as detected by LC/MS. ND = not detected.

	Intact	3′ n-1	3′ n-2	3′ n-3	3′ n-4	3′ n-5	3′ n-6
NHP Liver	71.3 ± 4.3	21.5 ± 4.9	1.0 ± 0.6	0.6 ± 0.1	1.5 ± 0.5	2.8 ± 0.2	1.5 ± 0.9
NHP Kidney	90.7 ± 3.0	9.3 ± 3.0	ND	ND	ND	ND	ND

All other metabolites observed were below 3% of total drug related material and thus excluded from further characterization. This data infers that the siRNA molecule evaluated herein is extremely stable *in vivo*, a known characteristic of fully chemically modified siRNA molecules, which contributes to the long duration of effect. The identification of the different metabolite patterns of synthetic siRNAs observed in the different tissues provides an insight into the stability and pharmacokinetic properties, in early discovery understanding the metabolite patterns can lead to the design of more stable, potent molecules. Although LC/MS can be powerful in identifying metabolite patterns in plasma and tissue, the sensitivity of LC/MS limits the applicability. Therefore, in most discovery settings, a more sensitive assay such as an ELISA, as reported here, must be utilized to measure siRNA concentrations in both plasma and in tissue.

### Quantification siRNA in biological matrices from non-human primates

**Figure 4:**
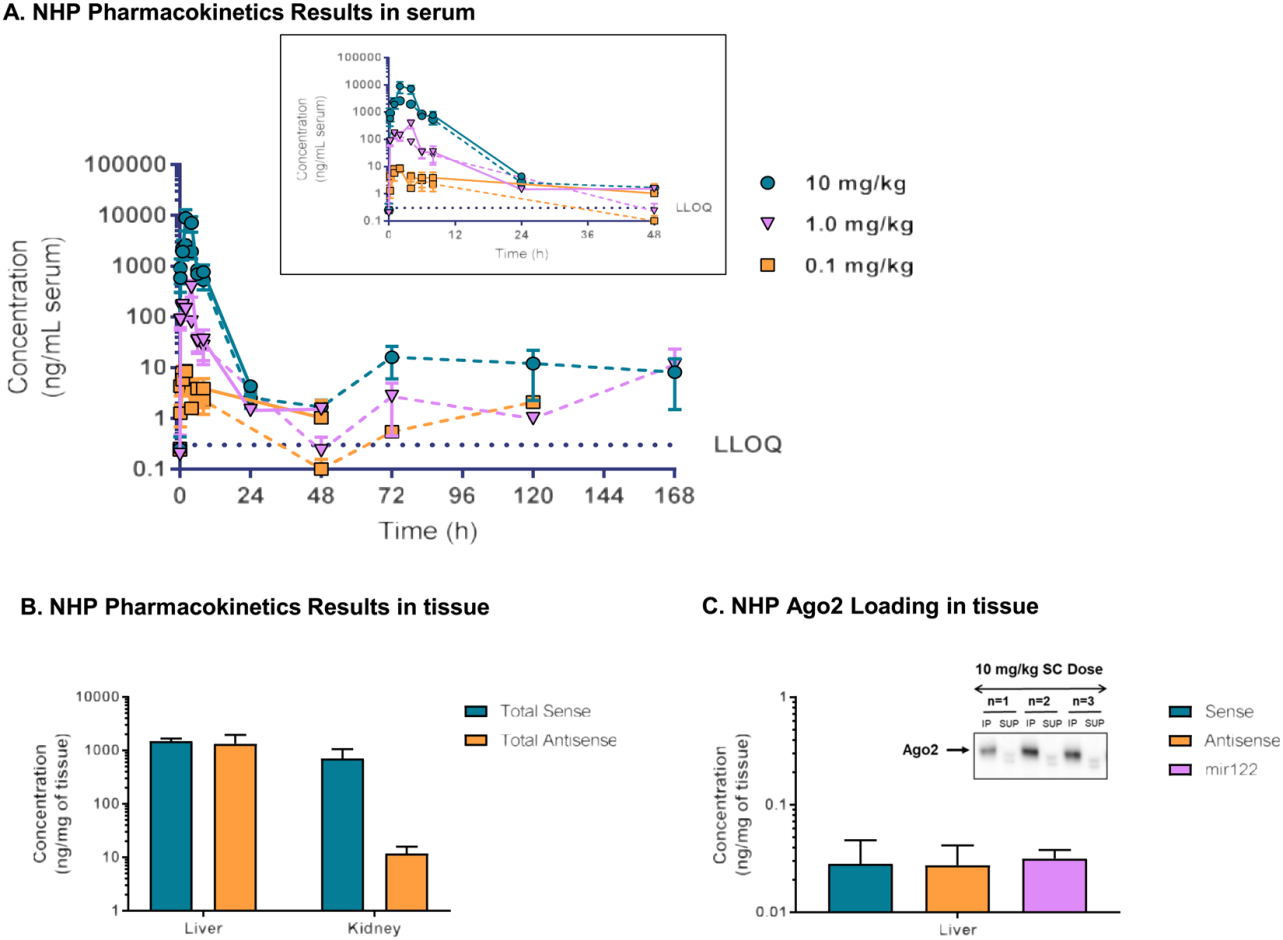
Measurement of siRNA molecule *in vivo*. (Serum exposures of siRNA molecule in NHPs (n = 6) after a single subcutaneous dose of with 0.1, 1 or 10 mg/kg. Solid lines represent sense strand and dashed lines represent antisense strand. Liver results 28 days post-dose (**B**). Ago2 pulldown in liver homogenate (n = 3) to measure siRNA amount bound (**C**). Cropped blot of pulldown displayed within inset (Full blot image provided in Supplementary Information).

Serum concentration-time profiles for sense and anti-sense strands of an siRNA duplex were first determined following a single subcutaneous (SC) dose siRNA at either 0.1, 1, or 10 mg/kg in NHPs (n = 6 animals per dosing group). Peak serum concentrations (Cmax) for both sense and antisense strands were dose proportional from 0.1 to 10 mg/kg doses. The maximum concentration of drug was observed 1 hour post-dose for all three treatments (Fig. 4, Table 3 and Supplementary Information). Rapid clearance of the total dose from the serum compartment was anticipated and is in line with previously published pharmacokinetic profiles of siRNA with SC dosing^38,39^. This is suggestive of inherent siRNA organ distribution which is reflected in the observed volume of distributions. Furthermore, from these data, we can infer that the siRNA duplex remains intact in NHP serum up to approximately 24 to 48 hours post-dose as both the sense and antisense strand concentrations did not differ significantly. Interestingly, the antisense strand persisted in detection the entire duration of serum sampling (168 hours) and is mediated by a currently unknown mechanism, whereas the sense strand fell below the limit of assay detection (0.3 ng/mL) at all time points exceeding 48 hours- independent of dose.

**Table 3:** Pharmacokinetic parameters of antisense strand shown as Mean (SD). AUCinf = area under the concentration time-curve from time 0 to infinity; Cmax = maximum serum concentration; t1/2 = terminal half-life; Tmax = time of maximum concentration; AUClast area under the concentration time-curve from time 0 to last measurable concentration; Vd/F volume of distribution; SC = subcutaneous. All values are reported to 3 significant digits, with the exception of Tmax, which is reported as median value.

Dose a (mg/kg)	Route	Tmax (mg/kg)	Cmax (ng/mL)	t1/2 (hr)	AUCinf (ng day/mL)	AUClast (ng hr/mL)	Vd/F (L/kg)
0.1	SC	1.0	8.04 (2.78)	3.13 (1.19)	51.4 (12.8)	39.7 (5.10)	101 (60.1)
1	SC	1.0	142 (38.4)	3.23 (3.11)	795 (269)	620 (145)	88.2 (8.00)
10	SC	1.0	2310 (1000)	4.39 (0.420)	16900 (3960)	16800 (3960)	40.2 (13.1)

Taking this work further, we investigated tissue exposure levels of siRNA, specifically to the liver and kidneys, following four SC doses at 10 mg/kg every seven days (Fig. 4). Similar concentrations of sense and antisense strands were observed in the liver 28 days post-dose at 1,490 and 1,340 ng/mg of tissue, respectively. In contrast, the sense strand (711.3 ng/mg tissue) accumulated in the kidneys at a concentration almost 60 times that of the antisense strand (11.8 ng/mg of tissue). This tissue-specific dichotomy suggests that the sense strand may have a faster rate of clearance compared to its counterpart, which may be further elucidated with a more refined pharmacokinetic study that includes multiple tissue time points. In addition, as the siRNA mechanism of action requires Ago2 duplex loading for target mRNA degradation, we then quantitated the amount of sense and antisense strands co-immunoprecipitated with the catalytic RISC enzyme from liver homogenate. These analytes were then measured in parallel with the endogenous microRNA mir122 as a qualitative assessment for protein enrichment across experimental replicates since this RNA remains tightly associated with Ago2 under basal conditions (Fig. 4C)^40^. A significant difference was not observed between sense (0.028 ng/mg of tissue) and antisense (0.027 ng/mg of tissue) strand Ago2 loading and concentrations were comparable to mir122 (0.032 ng/mg of tissue). Sense strand cleavage and release from Ago2 was most likely not observed under these multiple dosing conditions due to potential saturation of the enzyme’s binding capacity as a result of high dosing paradigm implemented in this study. Further, from these data, we observed that of the total siRNA liver exposure, less than 0.01% was bound to Ago2.

## Methods

### Materials

Sheep polyclonal anti-digoxigenin antibody (Roche, Cat. 11222089001) was conjugated with a ruthenium label using the MSD Gold Sulfo-Tag NHS-Ester conjugation kit (Meso Scale Diagnostics, Cat. R31AA-1). Standards and sample buffer were made as follows: 10 mM Tris-HCl [pH 8.0] and 1 mM EDTA. Hybridization buffer was made as follows: 60 mM Na2PO4 [pH 7.0, dibasic], 1 M NaCl, 5 mM EDTA, and 0.02% Tween 20. Ligation buffer was made as follows: 66 mM Tris-HCL [pH 7.6], 10 mM MgCl2, 10 mM DTT, 1 mM ATP, 5 U/mL T4 DNA Ligase (Invitrogen, Cat. 15224041). Nuclease buffer was made as follows: 3 mM ZnSO4, 100 mM NaCl, and 0.8 U/*μ*L S1 Nuclease (Thermo Fisher, Cat. 18001016). Lyophilized oligonucleotide probes were custom synthesized from Qiagen Inc. (Hilden, Germany). All chemicals and reagents were analytical grade or higher, if not specified. Metabolite-simulating 2OMe modified oligonucleotides (sequences not shown) and miR122 sequence were custom synthesized by Integrated DNA Technologies (Iowa, USA). Stock solutions of 100 *μ*M were prepared by reconstitution in molecular grade water. The sequences of all oligonucleotide probes, standards, and metabolites are shown below.

*miR122 standard*: 5′-rUrGrG rArGrU rGrUrG rArCrA rArUrG rGrUrG rUrUrU rG-3′

*miR122 capture probe*: 5′-TCG C+TC GTG CAA ACA+CCA TTG+TCA CAC+TCC A/3Bio/-3′

*antisense capture probe*: 5′-TCG C+TC GTG CAG+XXX XXX XX+X XXX X+XX CGA/3Bio/-3′

*sense capture probe*: 5′-TCG C+TC GTG TCG+XXX XXX XX+X XXX X+XX CTG/3Bio/-3′

*detection probe*: 5′-CA+CGAG+CGA/3Dig_N/-3′

*r(base)*: Ribonucleotide

+*(base)*: LNA modification

*3Bio*: Biotin Conjugation with C6 Linker

*3Dig_N*: Digoxygenin (NHS Ester)

### Hybridization-Ligation ELISA

Either duplex siRNA or single stranded controls were spiked into relative species tissue homogenate or serum matrix at a standard curve range of 2.6–166 pM (0.04–2500 ng/mL). The standard curves and samples were diluted 1:20 in sample buffer and added to a 96-well PCR plate to a final volume of 50 *μ*L. Sequence specific capture oligonucleotide was added to hybridization buffer to a final concentration of 50 nM and 50 *μ*L added to the samples in buffer. Sample and capture probe were hybridized on a thermal cycler under the following conditions: 90 °C for 5 minutes, 52 °C for 30 minutes, and a final hold at 12 °C. After hybridization, samples were transferred to a MSD GOLD 96-well Streptavidin SECTOR PR plate (Meso Scale Diagnostics, Cat. L13SA) for 30 minutes at room temperature. After incubation, the plate was washed with 1x wash solution (KPL, Cat. 50-63-00). Detection oligonucleotide was added to ligation buffer at 1 nM and the mix was added to the plate at a 50 *μ*L volume with subsequent incubation at 37 °C for one hour. After washing, 50 *μ*L of nuclease buffer was added to the wells. Specific to the sequences used, plates were incubated at 37 °C or room temperature for 30 minutes for tissue and serum samples, respectively. Plates were washed and incubated for 1 hour with 50 *μ*L of 2 *μ*g/mL ruthenium labeled anti-digoxygenin antibody in SuperBlock T20 TBS Blocking Buffer (Thermo Fisher, Cat. 37536). After a final wash, 150 *μ*L of MSD Read Buffer T (Meso Scale Diagnostics, Cat. R92TD) was added and the plate was read on a MSD Sector S 600 instrument (Rockville, MD, USA).

### Hybridization-Ligation Sample Preparation

Tissue samples were homogenized in lysis buffer containing 50 mM Tris HCl, 100 nM NaCl, 0.1% Triton X100, and protease inhibitor cocktail (Roche, Cat. 11836170001) to a final concentration of 200 mg/mL. Further dilution series of either tissue or serum was done in 5% tissue homogenate (200 mg/mL) or 5% serum, respectively in sample buffer.

### LC-MS Sample Preparation

Oligonucleotides were extracted from rat and NHP liver homogenates using the optimized solid phase extraction protocol on a Phenomenex Clarity OTX SPE 96-well plate (Cat. 8E-S103-EGA). Prior to use, the SPE plate was conditioned with 1 mL of methanol and equilibrated with 1 mL of 50 mM NaH2PO4, 2 mM NaN3, 10 mM K2EDTA in water (pH 5.5). The liver homogenate samples were mixed with 1 volume of 10% (v/v) H3PO4 for 10 minutes and then mixed with three volumes of Phenomenex lysis-loading buffer (Cat. AL0-8579) before being loaded on to the SPE plate at low pressure (approximately 2 psi). Then the SPE plate was washed with 1 mL of 50 mM NaH2PO4, 2 mM NaN3, 10 mM K2EDTA in water (pH 5.5) once, 1 mL of 50 mM NaH2PO4 in 50:50 water:acetonitrile (pH 5.5) four times, and 1 mL of 50 mM ammonium formate in 20:80 water:acetonitrile (pH 5.5) twice before eluted with 0.5 mL of 100 mM ammonium bicarbonate, 10 mM TCEP in 50:40:10 water:acetonitrile:THF (pH 9) twice. The eluents were then evaporated under N2 to approximately 200 *μ*L for LC-MS analysis.

### LC-MS Method and Analysis

Samples were subjected to LC-MS analysis on Agilent 1290 coupled with Thermo Scientific Obritrap Fusion Tribrid Mass Spectrometer (Waltham, Ma). Chromatographic separation was performed on Waters ACQUITY UPLC Oligonucleotide BEH C18 column: 130 Ångstrm, 1.7 *μ*m, 2.1 mm × 50 mm at 80 °C with a flow rate of 0.4 mL/min. Mobile phase A consisted of 15 mM triethylamine (TEA), 400 mM hexafluoroisopropanol (HFIP) in water, and mobile phase B consisted of the same concentrations of TEA and HFIP in methanol. Each sample (10 *μ*L) was injected and separated on the column using the following gradient: 2% B for the initial 0.5 minutes, followed by a linear gradient to 50% B at 9.5 minutes, then another linear gradient to 95% B at 10 minutes; kept at 95% for the next 2.5 minutes; and then ramped down to 2% B at 13 minutes and kept at 2% for 1 minutes. The mass spectrometer was operated in the negative ion mode at spray voltage of −3000 V and source fragmentation energy of 60 eV. Scan range was set as 600–2400 m/z and Obritrap resolution was set at 60 K. The Oligonucleotide Mass Assembler (OMA) module in the OMA and Oligonucleotide Peak Analyzer (OPA) free software package was used to first to generate a predictive list of m/z values for all the possible metabolites that might be formed from the full-length siRNA construct^41^. Then the OPA module was used to analyze a representative mass spectrometry data file to confirm which metabolites were actually observed in the sample. A processing method was then generated using the top three most intense charge states and the top three isotopes of each charge state to calculate the peak area of each observed metabolite.

### Nonhuman primate studies

All non-human primate studies were conducted at Charles River Laboratories (Reno, Nevada) or MPI Research Inc. (Manatwan, MI). Animals were housed in accordance with the USDA Animal Welfare Act (9 Code of Federal Regulations, parts 1, 2, and 3) and as described in the Guide for the Care and Use of Laboratory Animals. Studies were conducted under an approved IACUC protocol. For serum exposure analysis, female monkeys between 6–10 years of age were dosed subcutaneously with a dose of 0.1, 1.0, and 10 mg/kg. For tissue exposure analysis, female monkeys between 6–10 years of age were dosed at 10, 50, and 200 mg/kg once weekly for four weeks. Necropsy samples were taken 28 days post dose. Pharmacokinetic parameters were extrapolated from sense and antisense serum concentrations using WinNonLin non-compartmental analysis from time zero to 24 hours.

### Argonaute-2 (Ago2) Pulldown

Homogenized tissues (25 mg/mL) were centrifuged at 12,000 × g for 10 minutes at 4 °C to pellet cellular debris. The supernatant was then added to 25 *μ*L of either anti-mouse- or anti-human Ago2 antibody beads (Wako Chemicals, Cat. 292-67301 and 292-66701) that were pre-washed twice with 500 *μ*L of lysis buffer (50 mM Tris HCl, 100 mM NaCl, 0.1% Triton X100). Samples were incubated with Ago2 antibody beads overnight while gently rotating at 4 °C. Following incubation, beads were washed three times with 500 *μ*L of lysis buffer and immunoprecipitated protein was eluted with 100 *μ*L of Quantigene Plex lysis mixture (Affymetrix, Cat.10093) while boiling at 95 °C for 5 minutes^42^.

## Supplementary information

Supplemental Information
