# Supplementary material for: Application of Locked Nucleic Acid Oligonucleotides for siRNA Preclinical Bioanalytics
Source: Sci Rep. 2019 Mar 5;9:3566. doi: 10.1038/s41598-019-40187-4 (PMC6401054; doi:10.1038/s41598-019-40187-4)
Supplement: Supplementary file 1 — Supplemental Information [file 41598_2019_40187_MOESM1_ESM.docx]

**Application of Locked Nucleic Acid Oligonucleotides for siRNA Preclinical Bioanalytics**

# Mai B. Thayer^1,*^, Julie M. Lade^1^, David T. Doherty^1^, Fang Xie^1^, Babak Basiri^1^, Omar Barnaby^2^, Noor Bala^1^, and Brooke M. Rock^1,**^

^1^Amgen Research, Pharmacokinetics and Drug Metabolism, South San Francisco, 94080, USA

^2^Amgen Research, Pharmacokinetics and Drug Metabolism, Thousand Oaks, 91320, USA

^*^mthayer@amgen.com ^**^brooke@amgen.com

**Supplementary Information**

Supplemental Figures:


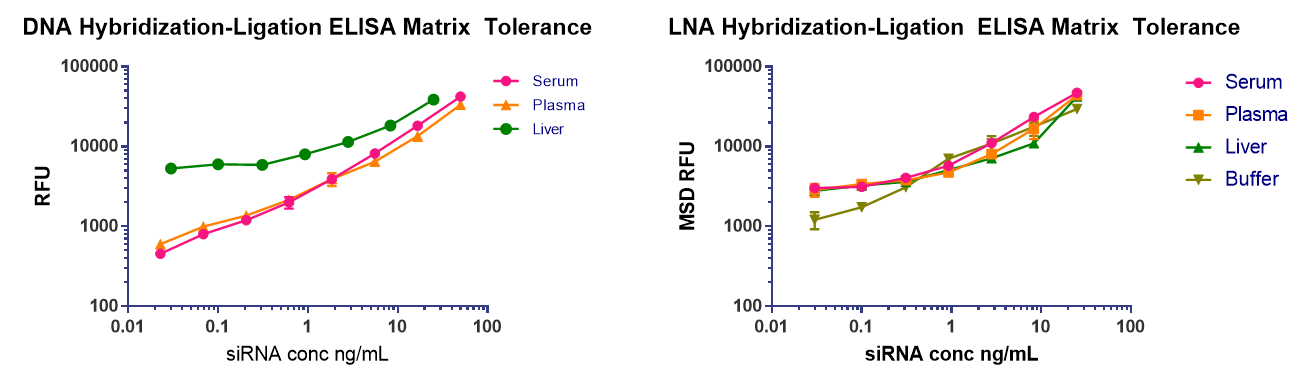


**Supplemental Figure 1.** Direct comparison of DNA (A) and LNA (B) modified oligonucleotide probes in the hybridization-ligation ELISA in rat serum, plasma, and tissue homogenate


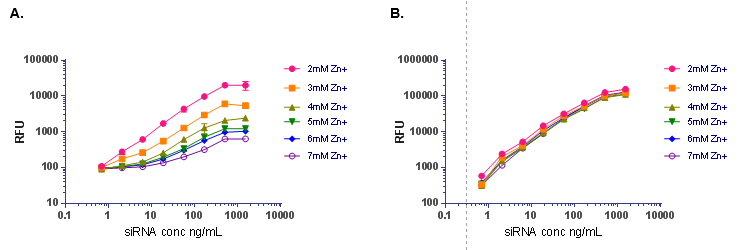


**Supplemental Figure 2.** Impact of Zn+ concentrations on the activity of S1 nuclease in the hybridization-ligation ELISA at 37°C (A) and room temperature (B). Assay curves prepared in sample buffer.


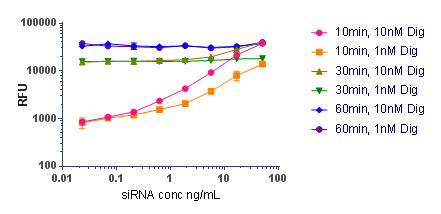


**Supplemental Figure 3.** Trends on nuclease activity in serum matrix at varying concentrations of 10 and 1nM digoxygenin conjugated detection oligonucleotide with time. Assay curves prepared in sample buffer.


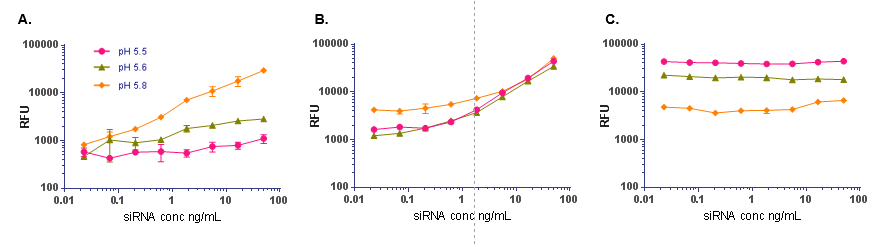


**Supplemental Figure 4.** Trends on nuclease activity in tissue homogenate at varying levels of pH (5.5-5,8) at 37°C after incubation times of 15 minutes (A), 30 minutes (B), and 1 hour (C) with constant amount S1 nuclease enzyme. Assay curves prepared in sample buffer.

|  |  | Mean (SD) | | | | |  |
| --- | --- | --- | --- | --- | --- | --- | --- |
| Dose^a^ (mg/kg) | Route | T_max_ (hr) | C_max_ (ng/ml) | t_1/2_ (hr) | AUC_inf_ (ng∙day/mL) | AUC_last_  (ng•hr/mL) | |
| 0.1 | SC | 2.0 | 8.65 (2.22) | 2.13 (1.67) | 42.3 (10.2) | 31.2 (4.56) | |
| 1 | SC | 2.0 | 383 (138) | 2.03 (0.981) | 654 (204) | 508 (120) | |
| 10 | SC | 2.0 | 9790 (2920) | 2.99 (0.670) | 15300 (2305) | 15100 (4320) | |

**Supplemental Table 1.** Pharmacokinetic parameters of sense strand shown as Mean (SD). AUC_inf_= area under the concentration time-curve for time 0 to infinity; C_max_= maximum serum concentration; t_1/2_= terminal half-life; T_max_= time of maximum concentration; AUC_last_= area under the concentration time-curve from time 0 to last measurable concentration; SC= subcutaneous. All values are reported to 3 significant digits, with the exception of T_max_, which is reported as median value.

|  | Intact | 3’ n-1 | 3’ n-2 | 3’ n-3 | 3’ n-4 | 3’ n-5 | 3’ n-6 |
| --- | --- | --- | --- | --- | --- | --- | --- |
| NHP Liver | 71.3 ± 4.3 | 21.5 ± 4.9 | 1.0 ± 0.6 | 0.6 ± 0.1 | 1.5 ± 0.5 | 2.8 ± 0.2 | 1.5 ± 0.9 |
| NHP Kidney | 90.7± 3.0 | 9.3±3.0 | ND | ND | ND | ND | ND |
| Rodent Liver | 13.0 ± 1.8 | 76.9 ± 1.5 | ND | 1.4 ± 0.2 | 4.9 ± 0.8 | 2.6 ± 0.4 | 1.2 ± 0.4 |
| Rodent Kidney | 39.2 ± 2.9 | 59.2 ± 3.0 | 0.3 ± 0.1 | 0.4 ± 0.2 | 0.4 ± 0.2 | 0.5 ± 0.1 | ND |

**Supplemental Table 2.** Percentage of intact anti-sense strand and metabolites in NHP and rodent liver and kidney samples measured by LC/MS. ND= not detected.


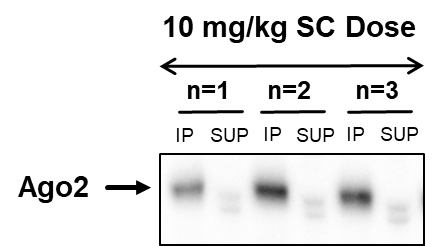

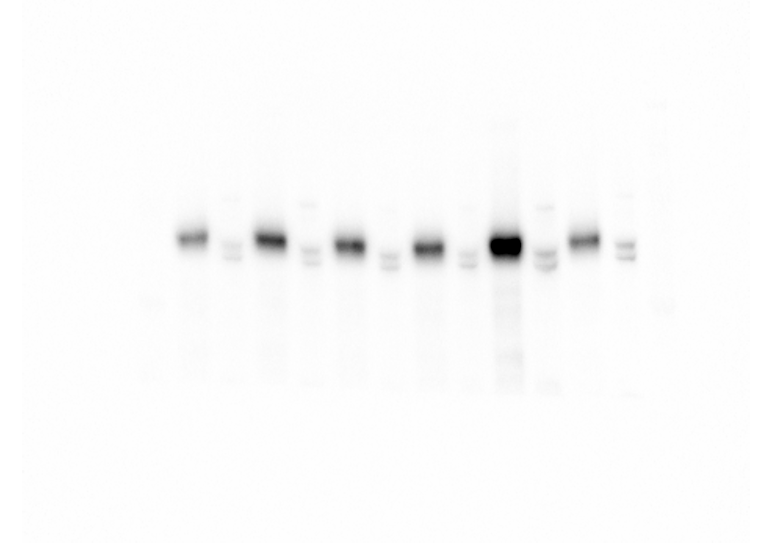


**Supplemental Figure 5.** Ago2 pulldown in NHP liver homogenate (n=3) to measure amount siRNA bound and corresponding full image of western blot. Inset images correspond to the first 6 lanes of blot. Remaining lanes 7-12 are data not discussed in this manuscript.
